# Secondhand Smoke: Displaced Enthusiasm?

**DOI:** 10.1289/ehp.115-a129a

**Published:** 2007-03

**Authors:** John Manuel

As evidence mounts that secondhand smoke (SHS) can harm human health, an increasing number of U.S. and Canadian cities are passing bans on smoking in restaurants and bars. Proposed bans have been opposed by a few commercial establishments and their respective trade associations, who fear they may lose clients as a result. Some heating, ventilating, and air-conditioning (HVAC) contractors have suggested that a method called “displacement ventilation” can effectively control SHS, making it unnecessary to impose smoking bans. But a recent study indicates these systems cannot be depended upon to bring SHS down to safe levels.

Displacement ventilation systems typically introduce fresh air at or near floor level at a temperature slightly below the desired room temperature. This cooler fresh air displaces the warmer room air at the occupied level; heat and pollutants rise to the ceiling and are drawn out by an exhaust fan. (In comparison, traditional HVAC systems supply air through ceiling vents and recirculate it after diluting it with outdoor air.) A study in the December 2001 issue of *Regulatory Toxicology and Pharmacology* reported that displacement ventilation can control SHS in smoking areas of restaurants. That study has been used to justify opposing local and provincial smoking ban proposals.

Citing various flaws in that study, James Repace, an adjunct professor of public health at Tufts University School of Medicine, and Kenneth Johnson, a research scientist with the Public Health Agency of Canada, undertook their own study of displacement ventilation, which was published in the fall 2006 issue of *IAQ Applications*. They selected the same establishment used in the 2001 study. The Black Dog Pub housed a smoking bar connected by two pass-through windows and two open doorways to a nonsmoking dining room. Ventilation air was drawn into the nonsmoking area and exhausted out the far corner of the smoking area.

Repace and Johnson conducted real-time measurements of particulate polycyclic aromatic hydrocarbons (PPAH), a tobacco smoke carcinogen, and respirable suspended particles (RSP), known to contribute to a variety of respiratory problems. The tests measured PPAH levels of 152 ng/m^3^ in the Black Dog’s smoking section and 16 ng/m^3^ in the nonsmoking section. RSP levels of 199 μg/m^3^ and 40 μg/m^3^ were recorded in the smoking and nonsmoking areas, respectively. Measurements taken later, after a smoking ban was implemented, showed that levels of RSP and PPAH dropped by 80% and 96%, respectively, in the smoking area, and by 60% and 80% in the non-smoking area. According to Repace, *de minimis* (i.e., negligible) risk levels of SHS would occur at average RSP concentrations of 0.075 ng/m^3^ for persons exposed to an average of 8 hours a day over 40 years (PPAH is not regulated).

The following year, Repace and Johnson conducted similar tests in two restaurants in Mesa, Arizona. The restaurants were exempt from the city’s nonsmoking ordinance based on their managers’ claims that they could meet smoke-free standards by using displacement ventilation. At Romano’s Macaroni Grill, RSP levels averaged 80 μg/m^3^ in the smoking bar and 229 μg/m^3^ in the adjacent nonsmoking restaurant. PPAH levels averaged 304 ng/m^3^ in the bar and 451 ng/m^3^ in the restaurant. At T.G.I. Friday’s, RSP levels averaged 205 μg/m^3^ in the smoking bar and 306 μg/m^3^ in the nonsmoking restaurant. PPAH levels averaged 13 ng/m^3^ in the bar and 2 ng/m^3^ in the restaurant (the latter reflects in part a period during which an outside door was propped open).

Based on the nonsmoking sections’ having higher levels of pollutants than the smoking sections, the authors concluded that the ventilation systems in both restaurants were seriously out of balance. However, the Black Dog system, though properly designed and operated, still could not prevent all workers and patrons from being exposed to hazardous levels of SHS.

David Sutton, a spokesman for Phillip Morris USA, says he can’t comment on displacement ventilation in particular, but maintains that “in many indoor public places, reasonable ways exist to respect the comfort and choices of both the smoking and nonsmoking adults.” Sutton says establishment owners “should have the flexibility to address the preferences of nonsmokers and smokers through separation, separate rooms, and/or high-quality ventilation.”

However, Repace and Johnson concluded that banning smoking is the only way to guarantee a smoke-free indoor environment. “The 2006 Surgeon General’s report states flatly that there is no safe level of SHS exposure,” Repace says. “Displacement ventilation is not a viable substitute for smoking bans in controlling SHS exposure in either designated smoking areas or in contiguous designated nonsmoking areas.” Repace says studies indicate that if you can’t smell tobacco smoke, you are probably not being exposed to a dangerous amount. However, he adds, people with heart conditions or asthma should avoid any place where people are smoking.

